# Left atrial appendage closure in patients with atrial fibrillation in whom warfarin is contra-indicated: initial South African experience

**DOI:** 10.5830/CVJA-2013-018

**Published:** 2013-06

**Authors:** Mark Abelson

**Affiliations:** Vergelegen Mediclinic, Somerset West, South Africa

**Keywords:** atrial fibrillation, warfarin, left atrial appendage plug

## Abstract

**Abstract:**

Atrial fibrillation is a common cause of cardiac embolic events, especially stroke. Oral anticoagulation therapy is used to reduce these events. Many patients however are unable to take such therapy. Percutaneous occlusion of the left atrial appendage (the source of 90% of these emboli) is an option in these patients. Presented here are the first 12 patients to have this procedure done in South Africa.

## Abstract

Atrial fibrillation (AF) is the most common cardiac arrhythmia.^1^ It is a major cause of morbidity and mortality due to not only cardio-embolic events such as stroke but also anticoagulant-related major bleeding complications.^2^ The prevalence of AF increases with age (affecting up to 15% of patients over 80 years) and in patients with predisposing conditions such as hypertension, diabetes, heart failure and ischaemic heart disease.^1^

Approximately 25% of ischaemic strokes are due to cardiac embolism because of underlying atrial fibrillation.^3^ Strokes in patients with atrial fibrillation are generally larger with a worse prognosis than in patients without atrial fibrillation.^4^ Anticoagulation with the vitamin K antagonist, warfarin, and the recently available new agents, dabigatran and rivaroxoban, are indicated to prevent cardiac thrombo-embolism from occurring in patients with atrial fibrillation and a CHADS_2_/CHADS-Vasc score > 1.^5^-^8^

In many patients, however, anticoagulation is contra-indicated due to high bleeding risk (HASBLED score > 3),^9^ life-threatening bleeds of unknown cause while on anticoagulation, or due to perceived frailty and high risk of falls, especially in very elderly patients.^10^,^11^ Furthermore, INR control of patients on warfarin is generally poor. This is particularly so in South Africa (South African patients enrolled in the Active W Trial had the worst INR control internationally, with < 50% of patients’ INR in the therapeutic range), possibly due to no-compliance or interactions with food, drugs and lifestyle.^12^

An alternative approach to prevention of cardiac embolism in patients with AF is therefore desirable. One that can be used in all patients and does not require anticoagulation other than low-dose aspirin. Post-mortem and trans-oesophageal echo studies have shown that approximately 90% of all cardiac thrombi originate from the left atrial appendage (LAA) in patients with AF.^13^ Surgical exclusion of the LAA at the time of mitral valve surgery or CABG has been shown to reduce the incidence of cardiac embolic events in patients with AF.^14^

It is on this basis that percutaneous exclusion of the LAA has been developed, initially using the Plato device^15^ (no longer in production) and followed by the Watchman (Boston Scientific)^16^ and the Amplatzer CardiacPlug (ACP) (St Jude) devices.^17^-^19^

This article reports the initial safety, feasibility and clinical follow up after ACP implantation for non-valvular AF in the first 12 patients to be operated on in South Africa.

## Methods

Twelve consecutive patients who received LAA occlusion with the ACP from November 2010 were prospectively studied. All patients had permanent atrial fibrillation of more than six months’ duration and a CHADS-Vasc score of > 2. All had a major contra-indication to anticoagulation. All but one patient had experienced at least one major bleed, requiring blood transfusion, despite a therapeutic INR. Patient characteristics are shown in Table 1. No patients were on warfarin at the time of the procedure. All patients had been pre-treated on the day of the procedure with aspirin 300 mg and clopidogrel 600 mg.

**Table 1:** Patient Characteristics

*Patient*	*Age*	*Gender*	*Type of AF*	*CHADS_2_-Vasc*	*Wafarin contra-indication*
1	73	Male	Permanent	5 (age, PVD, CCF, stroke)	Gastric bleed, labile INR
2	75	Male	Permanent	5 (age, HPT, stroke)	Stroke on warfarin, labile INR
3	75	Male	Permanent	5 (age, DM, IHD, CCF)	Major GI bleed
4	82	Male	Permanent	6 (age, stroke, HPT, PVD)	Recurrent major epistaxis
5	79	Male	Permanent	2 (age)	Recurrent major GI bleed
6	67	Male	Permanent	3 (age, HPT, IHD)	Major GI bleed
7	54	Male	Permanent	3 (DM, HPT, CCF)	Major GI bleed
8	61	Male	Permanent	3 (DM, HPT, IHD)	Major GI bleed
9	61	Male	Permanent	2 (HPT, CCF)	Active ulcerative colitis
10	62	female	Permanent	3 (female, stroke)	Labile INR
11	68	Male	Permanent	4 (age, HPT, IHD, DM)	Massive retroperitoneal bleed
12	65	Male	Permanent	4 (age, HPT, stroke)	Spontaneous subdural haemorrhage

Ten procedures were done at Vergelegen Medi-Clinic, Somerset West. The first seven were with proctor guidance. One was done at Groote Schuur Hospital, Cape Town and one at Vincent Pallotti Hospital, Cape Town. All procedures were done under general anaesthetic, as transoesophageal echocardiogram (TOE) guidance is required during the procedure. Trans-septal puncture was done via the right femoral vein in the usual manner using TOE guidance. Following trans-septal puncture, 5 000 units of intravenous heparin was given and ACT was maintained > 250 s for the procedure.

An angiogram was then taken of the left atrial appendage to size the left atrial appendage orifice. A suitably sized device was then selected and placed in the appropriate position using TOE and angiographic guidance. All patients had a transthoracic echocardiogram after the procedure and the next day to exclude a pericardial effusion or device shift.

Patients were discharged home the following day on aspirin 82–150 mg daily indefinately and clopidogrel 75 mg daily for a month. All patients were seen at one month post procedure with a transthoracic echocardiogram, and six-monthly thereafter.

## Results

There were no procedure-related complications and 100% implantation success was achieved. In one case the initial device chosen was too small and a second larger device was chosen (Table 2, procedure characteristics). The average device size was 25.8 mm. All patients were disharged home the following day.

**Table 2:** Procedure Characteristics (*n* = 12)

Implantation success	12
Residual leak	1
Device size	25.8 mm (22–30)
Fluoroscopy time	23.14 min (14.9–35.4)
Hospital stay duration	1 day in all patients

There were no serious safety events, particularly pericardial effussion, device embolisation or procedure-related stroke (Table 3). No patients had pericardial effusions seen on echo immediately after the procedure and on discharge the following day.

**Table 3:** Complications – Acute And On Follow Up (*n* = 12)

Cardiac tamponade	0
Pericardial effusion	0
Device embolisation	0
Procedure-related stroke	0
Subsequent stroke/embolic event off warfarin	0
Access site complications	0
Death, all cause	0
Duration of follow up (mean)	12.8 months (2–20)

Clopidogrel was stopped in all patients at one month, except one patient who stopped the clopidogrel after one week due to recurrent epistaxis (Patient 4). On follow up varying between three and 20 months, no cardiac embolic events had been recorded in any patients. All devices are well seated and there have been no device-related complications.

## Discussion

Occluding the LAA in patients with AF and thereby preventing the vast majority of intracardiac thrombus formation is a highly attractive concept, especially for those patients who are unable to take any form of oral anticoagulant therapy. The patients, however, must be made aware that exclusion of the LAA, similar to when using warfarin, does not absolutely exclude risk of future strokes.

The Protect AF study,^16^ using the Watchman device, randomised 542 AF patients, 2:1 between the device versus warfarin. This study showed the device was non-inferior to warfarin in terms of stroke prophylaxis with a trend towards superiority. In the successfully treated population (device deployed and warfarin stopped) the primary efficacy event rate (all stroke) in the intervention group who discontinued warfarin was 1.9 per 100 patient years compared with 4.6 per 100 patient years in the control group who received warfarin (RR = 0.40).

Primary safety events occurred at a higher rate in the intervention group than in the control group (7.4 per 100 patient years vs 4.4 per 100 patient years; RR = 1.69). The most frequent primary safety event in the intervention group was serious pericardial effusion (requiring drainage), which occurred in 4.8% of patients. No patients with pericardial effusion died. Most safety events occurred during the first three implant procedures (12.3 vs 5.9% subsequently). These safety events have been further significantly reduced in the subsequent 460 patients enrolled in the Continued Access Patient Registry to 3.7%, most likely due to increasing operator experience.^20^

**Fig. 1.**
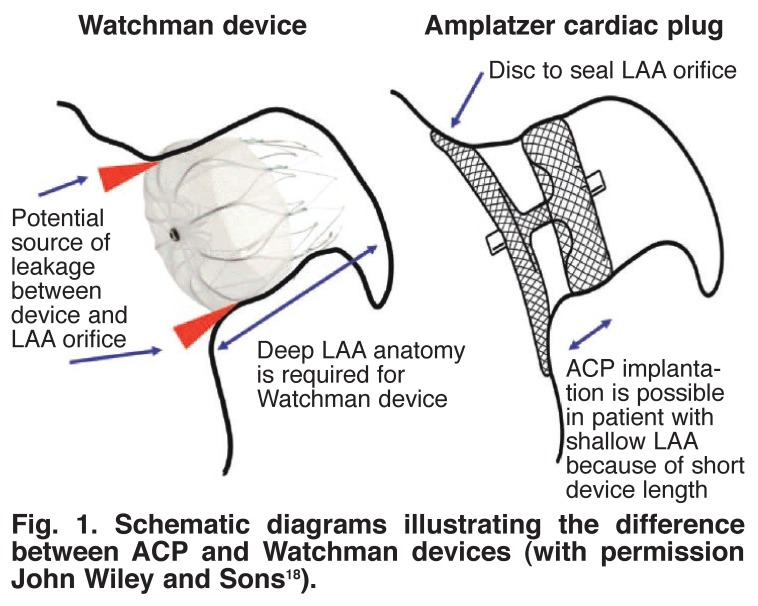
Schematic diagrams illustrating the difference between ACP and Watchman devices (with permission John Wiley and Sons^18^).

**Fig. 2.**
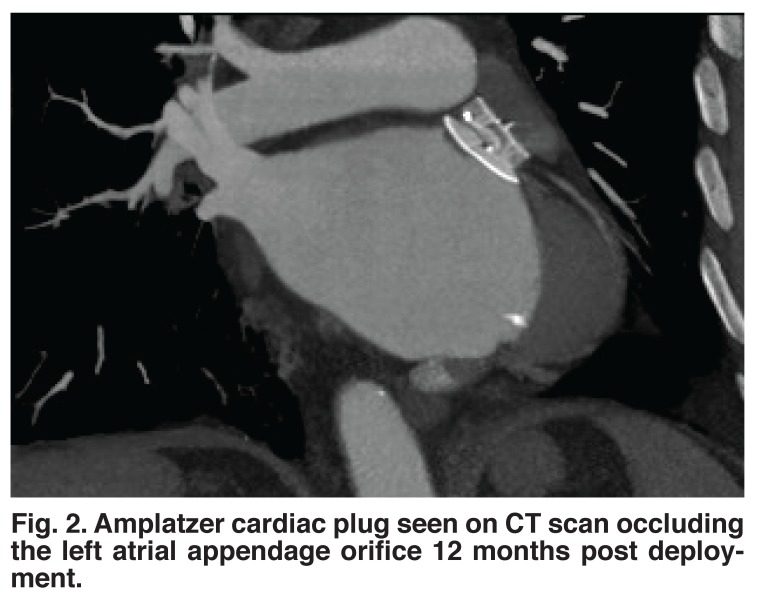
Amplatzer cardiac plug seen on CT scan occluding the left atrial appendage orifice 12 months post deployment.

The ACP is used extensively in Europe and Asia as an alternative to the Watchman device due to it’s perceived superiority in sealing the LAA orifice (Figs 1, 2). The Watchman device consists of a parachute-shaped nitonol frame which plugs the LAA orifice while the ACP consists of two parts; one, the lobe, fixes the device into the left atrial appendage and second, the disc, which seals off the LAA orifice. The incidence of leaks into the LAA on follow-up TEE is approximately 30% with the Watchman device and just over 1% with the ACP.^19^

The European Prospective Observational Study (*n* = 204) using the ACP in patients with AF who have contra-indications for oral anticoagulant therapy had a 96.6% implantation success rate with a total safety event rate of 2.9% (serious pericardial effussion 1.5%). There were no procedure-related strokes or TIA. After 101 patient years’ follow up, the actual stroke rate was 1.98%. The estimated annual stroke risk was 5.6%, according to the average CHADS2 score of 2.6.^19^

Although randomised, controlled trials comparing the ACP device versus either warfarin or no anticoagulant treatment are still currently in progress, observational studies have shown that the actual incidence of stroke is less than half of the predicted risk using the CHADS_2_ score when used in patients in whom warfarin is contra-indicated.^17^-^19^

In the 12 South African patients presented here, nine had had at least one major bleed requiring blood transfusion despite a sub-therapeutic/therapeutic INR level. No bleeds were related to warfarin toxicity. The average CHADS_2_-Vasc score was 3.75, which gives an estimated annual stroke risk of nearly 4% in patients who are unable to take warfarin. The implantation success rate was 100%. There were no safety events – in particular no stroke or TIA, no device embolisation and no pericardial effusion.

All patients are currently on low-dose aspirin alone and there have been no strokes or other cardio-embolic events to date. There have been no deaths of any cause. No patients have had any further major bleeds after stopping warfarin therapy.

## Conclusion

In patients with AF and a CHADS2-Vasc score > 2 who are unable to take oral anticoagulant therapy, percutaneous occlusion of the LAA is a reasonable option to consider. As more data regarding this procedure become available over time and the results confirm the current positive results, this procedure could potentially become a first-line consideration in patients with AF facing a lifetime of anticoagulation.
